# Cost of TB services in the public and private sectors in Georgia

**DOI:** 10.5588/ijtld.21.0176

**Published:** 2021-12-01

**Authors:** I. Chikovani, N. Shengelia, N. Marjanishvili, T. Gabunia, I. Khonelidze, L. Cunnama, I. Garcia Baena, N. Kitson, S. Sweeney, A. Vassall, Y. V. Laurence

**Affiliations:** 1 Curatio International Foundation, Tbilisi, Georgia; 2 Ministry of Internally Displaced Persons from the Occupied Territories, Labour, Health and Social Affairs, Tbilisi, Georgia; 3 National Center for Disease Control and Public Health, Tbilisi, Georgia; 4 Health Economics Unit & Health Economics Division, School of Public Health and Family Medicine, University of Cape Town, Cape Town, South Africa; 5 TB Monitoring and Evaluation, Global TB Programme, World Health Organization, Geneva, Switzerland; 6 Centre for Health Economics in London, Department of Global Health and Development, London School of Hygiene & Tropical Medicine, London, UK

**Keywords:** tuberculosis, unit costs, top-down approach, financing, Georgia

## Abstract

**BACKGROUND:**

Patient-centred care along with optimal financing of inpatient and outpatient services are the main priorities of the Georgia National TB Programme (NTP). This paper presents TB diagnostics and treatment unit cost, their comparison with NTP tariffs and how the study findings informed TB financing policy.

**METHODS:**

Top-down (TD) and bottom-up (BU) mean unit costs for TB interventions by episode of care were calculated. TD costs were compared with NTP tariffs, and variations in these and the unit costs cost composition between public and private facilities was assessed.

**RESULTS:**

Outpatient interventions costs exceeded NTP tariffs. Unit costs in private facilities were higher compared with public providers. There was very little difference between per-day costs for drug-susceptible treatment and NTP tariffs in case of inpatient services. Treatment day financing exceeded actual costs in the capital (public facility) for drug-resistant TB, and this was lower in the regions.

**CONCLUSION:**

Use of reliable unit costs for TB services at policy discussions led to a shift from per-day payment to a diagnosis-related group model in TB inpatient financing in 2020. A next step will be informing policy decisions on outpatient TB care financing to reduce the existing gap between funding and costs.

## Abstract (fr)

**CONTEXTE:**

Des soins centrés sur le patient et un financement optimal des services fournis aux patients hospitalisés et ambulatoires sont les principales priorités du programme national de lutte contre la TB (NTP) en Géorgie. Cette publication présente le coût unitaire du traitement et celui du diagnostic de la TB, les compare aux tarifs fixés par le NTP et décrit comment les résultats de l’étude ont éclairé les politiques de financement de la TB.

**MÉTHODES:**

Les coûts unitaires moyens, évalués par méthodes descendante (TD) et ascendante (BU), des interventions antituberculeuses ont été calculés par épisode de soins. Les coûts TD ont été comparés aux tarifs fixés par le NTP. Leurs variations et celles de la composition des coûts unitaires entre centres publics et privés ont été analysées.

**RÉSULTATS:**

Le coût des interventions ambulatoires était supérieur aux tarifs fixés par le NTP. Les coûts unitaires dans les centres privés étaient plus élevés que dans les centres publics. Très peu de différences ont été observées entre les coûts journaliers du traitement de la TB pharmacosensible et les tarifs fixés par le NTP pour les services fournis aux patients hospitalisés. Le financement journalier du traitement dépassait les coûts réels dans la capitale (centres publics) pour la TB pharmacorésistante, et était plus faible en région.

**CONCLUSION:**

L’utilisation de coûts unitaires fiables pour les services antituberculeux lors de discussions politiques a conduit à abandonner la tarification à la journée pour un modèle de tarification à l’activité pour le financement des soins antituberculeux des patients hospitalisés en 2020. La prochaine étape serait d’éclairer les décisions politiques relatives au financement des soins antituberculeux ambulatoires pour réduire l’écart existant entre financement et coûts.

Prior to 2016, Georgia was among one of the high burden countries for drug-resistant TB (DR-TB), but has since achieved significant progress in its TB response.^1^ The incidence rate for all forms of TB dropped from 99 to 74 per 100,000 population (range 62–67) during 2015–2019.^2^,^3^ With 2,169 notified TB cases in a population of 3.7 million people in 2019, TB drug resistance remains a key challenge for the National TB Programme (NTP). The proportion of rifampicin-resistant/multidrugresistant TB (RR/MDR-TB) remains at 12% (range 10–14) among new cases, but dropped from 39% to 32% (range 28–37) among previously treated cases since 2015.^2^,^3^

TB services are delivered by a mix of public and private service providers. Specialised TB public facilities are concentrated in Tbilisi City and a couple of other urban areas, while in the districts and regional centres there is a network of stand-alone private facilities where TB services are integrated into general health care.

The traditional financing approach of fixed tariffs per bed-day for drug-susceptible (DS-) or drug-resistant (DR-) TB inpatient care has created a perverse incentive for hospitalisation for diagnosis and DS-TB treatment, and prolonged hospital stays. Although this trend has decreased over the last decade,^4^,^5^ 25% of DS-TB and 80% of DR-TB patients were hospitalised for respectively 30 and 60 days in 2017–2018.^3^

Guided by the National TB Strategy for 2019–2020, and in line with the WHO Global END TB Strategy,^6^ Georgia is moving towards a patient-centred approach by reducing hospitalisations and shifting to the outpatient care model.^7^ Outpatient care is financed through case-based payment for TB diagnostics and contact screening, and monthly DS- or DR-TB vouchers for institutions per patient treated.

Improving the efficiency of TB financing has become especially important during the transition away from Global Fund support. To address inefficiencies, the Ministry of Internally Displaced persons from the Occupied Territories, Labour, Health and Social Affairs (MoILHSA) and the National Center for Disease Control and Public Health (NCDC) commissioned a project to develop policy recommendations on optimal financing of TB hospital care by 2020.

Implementation of the policies around patient-centred care and optimal financing of inpatient and outpatient care require accurate and current unit cost estimates for TB services,^7^ which were lacking in Georgia. Value TB, a Bill and Melinda Gates Foundation (Seattle, WA, USA) funded multi-country study to estimate the unit costs of TB services from the health service providers’ perspective, was conducted in Georgia in 2019. This paper presents unit cost estimates derived from that study, compares Value TB unit costs with the budgeted NTP values (tariffs) for diagnostics and treatment, and illustrates how Value TB findings informed policy discussions around optimising investments in TB care. The paper also presents cost variations and cost drivers for further policy discussions.

## METHODS

The study methods were adapted from “Costing guidelines for tuberculosis interventions” and the “Value TB” protocol template.^8^ Costs were estimated from a health provider’s perspective. Full financial and economic costs were collected retrospectively and reflected ‘real world’ implementation of TB interventions, but excluded surgical interventions as not within the scope of this study according to the above protocol. The time horizon was one patient episode of care. No start-up costs or costs of supporting change (for example, costs of piloting new interventions) were included. Estimation of future savings, above service level costs, research costs and other unrelated costs were also excluded.

### Sampling

The sampling frame consisted of the total list of Georgian healthcare facilities offering active and passive case-finding, diagnostic tests, and outpatient and inpatient services for TB, with some facilities providing more than one type of service (*n* = 133 sites). The sample size estimation was pragmatic based on budget availability. Facilities were selected using different criteria. Laboratories, public health centres, rural facilities providing only directly observed TB treatment (DOT), and outpatient facilities selection was random, proportional to size. The National Reference Laboratory was purposively selected, and inpatient facilities were selected based on bed days. The rural TB DOT facilities were excluded from the analysis for this paper because DOT services were captured through outpatient facilities included in the study, resulting in 27 sites or 22 facilities (see Supplementary Tables S1 and S2 for details). Sample weights (total patients or bed days and public or private ownership) were used to adjust for differences in probability of selection.

### Data collection

Data were collected for the 2018 financial year in Georgian lari (GEL) and converted into US dollars at the mean 2018 exchange rate of USD1 = GEL2.53 using a standardised Microsoft Excel tool (Microsoft, Redmond, WA, USA)—the Value TB Costing Tool Suite—adapted to the country context by four enumerators during January–May 2019. TB services and intervention unit costs represent existing practice and costs for 2018. Capital and recurrent prices were obtained from facilities’ financial departments, the NCDC (for centrally procured or donated goods and services) and Georgian market sources (see Supplementary Tables S3 and S4 for more details on price sources, allocation methods and assumptions). A local discount rate of 3% was used to annuitize capital goods with a useful life longer than 1 year. As direct observation was not possible for bacteriological and radiology tests due to access restrictions, timesheets were used to estimate staff time for these services.

Ethical approval was obtained from the Institutional Ethics Committees of the NCDC, Tbilisi, Georgia (Ref. 2019-030) and the London School of Hygiene & Tropical Medicine, London, UK (Ref. 17156). We obtained informed consent from all persons interviewed and observed.

### Costing approach, analysis and TB budgeting

The unit costs for TB interventions included in Value TB were obtained using both the top-down (TD) and bottom-up (BU) costing approaches. At each sampled facility, the costs of capital assets, staff and recurrent costs (including overhead, consumables and drugs) were identified, measured and valued for each TB service output. The appropriate service output unit costs, including outpatient visits, inpatient ‘hotel’ bed days, support services, and diagnostic and monitoring tests, were then combined to produce the unit costs per episode of care (TB detection and diagnosis, prevention, first-line and second-line treatment by phases).

Value TB costs were then used to help revise provider payments for TB services. To inform policy makers, we first estimated the national average unit cost. The latter represent a weighted mean based on the total number of patients receiving outpatient TB treatment and public/private ownership, and total bed days for IP DS-TB care at the respective facilities. No weighting was applied for inpatient DR-TB care unit costs, as all three facilities providing inpatient services for DR-TB patients were included in the costing.

We then compared weighted mean unit costs with budgeted NTP tariffs to support the assessment of the incremental financing requirements of adjusting provider payments to reflect the costs of TB services. In order to compare directly, we needed to adjust Value TB unit costs to be in line with the elements in the current provider payment covering diagnostics vouchers, outpatient DS-TB and DR-TB treatment monthly vouchers, and inpatient DS-TB and DR-TB bed days. We removed from Value TB unit costs the remaining cost components that are funded from other sources and are not part of above mentioned payments to providers (TB drugs and tests for Xpert^®^ MTB/RIF [Cepheid, Sunnyvale, CA, USA], drug susceptibility testing [DST], culture, HIV and hepatitis C virus [HCV] testing). As it was unclear whether to use the TD or BU cost, we first analysed the difference in TD and BU costs using a paired *t*-test.

After presenting estimates to key policymakers at the MolLHSA and NCDC, it was determined that TD costs would be more suitable for further policy discussions around TB service financing models. This would enable sustainability of financing in the short run as payment systems transitioned.

In 2019, the MoILHSA re-evaluated the budgeted NTP tariffs for outpatient TB interventions to be used in the following fiscal year, and increased salary component by 30% and 35% for outpatient diagnostic and treatment services to bring them closer to the average salary of family physicians. As the Value TB study estimated unit costs for 2018, these did not reflect the 2019 salary increase. Therefore, in order to compare the Value TB unit costs with the revised NTP budgeted tariffs, staff salaries were similarly increased for unit costs at facilities offering outpatient TB services. As the NTP did not envision salary increases for hospital interventions in 2019, no salary adjustment was done for inpatient TB services. Finally, we adjusted for inflation since the year of data collection and our TD unit costs for outpatient services were increased by respectively 4.97% and 4.7% for 2019 and 2020.^9^

The variations in, and cost composition of the TD unit costs between public and private facilities were then assessed, and this information was also provided to policy makers in setting the final price for TB services.

Stata SE v16.1 (Stata, College Station, TX, USA) was used to pool and clean data, and to create summary descriptive tables of unit costs by approach (TD vs. BU) and by input. Data were exported to Microsoft Excel (Microsoft). SPSS v23.0 (IBM Corp, Armonk, NY, USA) and Stata SE v16.1 were used for cost comparisons.

## RESULTS

### Value TB unit costs of TB interventions: top-down and bottom-up approaches

Tables 1–3 present the TD and BU Value TB weighted mean unit costs for TB interventions by episode of care for pulmonary TB (PTB) and extrapulmonary TB (EPTB) case detection and diagnosis using active and passive case-finding; TB prevention; and first- and second-line TB treatment for adult and child PTB and EPTB. Value TB unit costs of TB services included in the interventions are detailed in Supplementary Tables S5–S7 and in Dataverse.^10^

**Table 1:** Value TB weighted mean unit costs by intervention (top-down and bottom-up), in 2018 USD^ * † ‡
^

Intervention type	Top-down weighted mean intervention cost mean (min–max) USD	Bottom-up weighted mean intervention cost mean (min–max) USD
TB case detection and diagnosis		
Active case-finding		
Health facility: FAST^§^	61.08 (19.02–104.01)	20.24 (15.34–22.69)
Health facility: contact visit	18.36 (9.1–35.92)	10.41 (6.1–14.74)
Contact tracing: epidemiologist^¶^	16.64 (13.64–19.63)	11.29 (10.02–12.55)
Passive case-finding^#^		
Adult PTB	226.13 (210.56–310.36)	146.84 (140.24–156.14)
Adult EPTB	10.67 (4.27–33.33)	5.89 (1.77–27.17)
Child PTB	221.22	156.15
TB prevention		
Child aged <5 years contact – HIV: 3HR	9.99	8.35
Child aged <5 years contact – HIV: 6H	15.6	8.96

^*^ Cost per patient per episode of care.

^†^Includes the following cost categories: 1) capital cost: buildings, laboratory and medical equipment, other equipment, furniture, vehicles, training; 2) recurrent costs: clinical and support staff, medical supplies, drugs, other non-medical supplies, capital maintenance, utilities, fuel and other transport recurrent, including maintenance and courier services, food, supplements, including food services, other recurrent.

^‡^Weighted means were estimated to reflect national average and were based on sample weights.

^§^An intervention when a health care provider (family doctor or nurse) in general health care facility identifies the self-reporting patients with productive cough and refers them to the Xpert testing. The cost excludes family doctor and family nurse costs.

^¶^Unweighted mean.

^#^Include all diagnostic tests, costs of the TB-specific laboratory tests (culture, DST, LPAs) derived from the national level are added.

USD =US dollars; PTB=pulmonary TB; EPTB=extrapulmonary TB; 3HR=3 months of daily isoniazid plus rifampicin; 6H = 6 months of daily isoniazid treatment; DST = drug susceptibility testing; LPA = line-probe assay.

**Table 2:** Value TB weighted mean unit costs for first-line treatment by treatment phase (top-down and bottom-up), in 2018 USD^ * † ‡
^

TB intervention/unit	Top-down weighted mean intervention cost mean (min-max) USD		Bottom-up weighted mean intervention cost mean (min-max) USD	
	
	Intensive phase	Continuation phase	Intensive phase	Continuation phase
Adult EPTB: new and relapse	212.34 (66.61–648.78)	186.67 (136.95–231.29)	130.06 (28.83–323.12	107 (68.34–173.16)
Adult EPTB: previously treated	359.61 (97.74–648.78)	245.89 (211.54–292.98)	226.35 (78.41–323.12)	181.3 (94.71–258.86)
Adult PTB: new and relapse^§^	222.61 (110.8–762.29)	265.25 (156.75–537.52)	128.57 (63.38–585.45)	131.64 (80.77–206.74)
Adult PTB: previously treated^§^	322.58 (149.42–762.29)	317.09 (266.18–412.26)	198.65 (92.81–598.09)	205.93 (128.78–292.44)
Child EPTB: new and relapse	198.94	104.31	178.04	90.12
Child EPTB: previously treated	198.94	104.31	178.04	90.12
Child PTB: new and relapse	521.22 (444.78–597.66)	163.97 (125.63–202.32)	342.23 (281.33–403.14)	96.84 (86.99–106.68)
Child PTB: previously treated	444.78	125.63	403.14	106.68

^*^ Cost per patient per episode of care.

^†^Includes the following cost categories: 1) capital cost: buildings, laboratory and medical equipment, other equipment, furniture, vehicles, training; 2) recurrent costs: clinical and support staff, medical supplies, drugs, other non-medical supplies, capital maintenance, utilities, fuel and other transport recurrent, including maintenance and courier services, food, supplements, including food services, other recurrent.

^‡^Weighted means were estimated to reflect national average and were based on sample weights.

^§^Includes all diagnostic tests, costs of the TB-specific laboratory tests (culture, DST, LPAs) derived from national-level data.

USD = US dollars; PTB = pulmonary TB; EPTB = extrapulmonary TB; DST = drug susceptibility testing; LPA = line-probe assay.

**Table 3:** Value TB weighted mean unit costs for second-line treatment by treatment phases (top-down and bottom-up) in 2018 USD^ * † ‡
^

TB intervention/unit	Top-down weighted mean intervention cost Mean (min–max)			Bottom-up weighted mean intervention cost Mean (min-max)		
	
	Intensive inpatient USD	Intensive outpatient USD	Continuation outpatient USD	Intensive inpatient USD	Intensive outpatient USD	Continuation outpatient USD
Adult MDR-TB, EPTB, long regimen	1,624.83^§^	1,879.23 (1,484.47–2,238.1)	3,500.57 (3,020.02–3,937.43)	716.29	1,428.64 (811.96–1,989.26)	2,637.29 (1,671.76–3,515.05)
Adult MDR-TB, PTB, long regimen	2,838.72^§¶^ (1,673.36–3,967.88)	3,306.34^¶^ (2,221.58–5,057.81)	5,696.15^¶^ (3,601.42–9,112.79)	2,304.39^§¶^ (1,185.76–3,486.56)	2,465.63^¶^ (1,880.56–3,085.82)	4,327.48^¶^ (3,207.39–5,590.85)
Adult, pre-XDR-TB, EPTB	873.91^§^	2,255.61	3,989.62	713.48^§^	1,999.29	3,548.1
Adult, pre-XDR-TB, PTB	4037.35^§^	2,188.41	4,189.01	3,550.36^§^	1,900.86	3,676.76
Adult, XDR-TB, EPTB	873.91^§^	2,255.61	3,989.62	713.48^§^	1,999.29	3,548.1
Adult, XDR-TB, PTB	4,037.35^§^	3,133.06^¶^ (2,199.12–4,276.5)	5,436.45^¶^ (3,578.96–7,669.84)	3,550.36^§^	2,401.46^¶^ (1,865.57–2,907.66)	4,239.73^¶^ (3,192.4–5,220.63)
Child, MDR-TB, EPTB, long regimen	1,438.32^§^	2,204.34	3869.57	618.17^§^	1,961.46	3,459.51
Child, MDR-TB, PTB, long regimen	3,695.45^§^	2,089.62	3,965.02	3,344.79^§^	1,834.83	3,521.53
Child, pre-XDR-TB, EPTB	691.77^§^	2,204.34	3,877.98	618.17^§^	1,961.46	3,467.47
Child, pre-XDR-TB, PTB	3,625.52^§^	2,089.62	3,991.28	3,279.89^§^	1,834.83	3,536.58
Child, XDR-TB, EPTB	718.04^§^	2,230.61	3,930.51	633.22^§^	1,976.51	3,497.57
Child, XDR-TB, PTB	3,718.77^§^	2,089.62	3,991.28	3,366.42^§^	1,834.83	3,536.58

^*^ Cost per patient per episode of care.

^†^ Includes the following cost categories: 1) capital cost: buildings, laboratory and medical equipment, other equipment, furniture, vehicles, training; 2) recurrent costs: clinical and support staff, medical supplies, drugs, other non-medical supplies, capital maintenance, utilities, fuel and other transport recurrent, including maintenance and courier services, food, supplements, including food services, other recurrent.

^‡^ Weighted means were estimated to reflect national average and were based on sample weights.

^§^ An intervention when a health care provider (family doctor or nurse) in general health care facility identifies the self-reporting patients with productive cough and refers them to the GeneXpert testing. The cost excludes family doctor and family nurse costs.

^¶^ Include all diagnostic tests, costs of the TB-specific laboratory tests (culture, DST, LPAs) derived from national-level data.

USD = US dollars; MDR-TB = multidrug-resistant TB; EPTB = extrapulmonary TB; PTB = pulmonary TB; XDR-TB = extensively drug-resistant; DST = drug susceptibility testing; LPA = line-probe assay.

Table 4 shows the differences in weighted mean unit costs between TD and BU unit costs approaches where intervention composition is aligned with NTP tariffs (see Methods). The estimated TD costs are between 34% (DR-TB treatment per day) and 132% (monthly outpatient DR-TB treatment continuation phase) higher than BU costs for all interventions. These differences are significant (*P* < 0.01) for all interventions, except for the second phase outpatient MDR-TB treatment and inpatient DR-TB bed day.

**Table 4:** Comparison of top-down and bottom-up Value TB weighted mean intervention unit costs, in 2018 GEL and USD

TB intervention^*^	Top-down		Bottom-up		*P* value
	
	Weighted mean unit cost (GEL)	Weighted mean unit cost (USD)	Weighted mean unit cost (GEL)	Weighted mean unit cost (USD)	
Outpatient services					
PTB detection and diagnosis					
Risk group screening: active PTB	58.95 (33.47–90.22)	23.29 (13.22–35.64)	32.51 (22.49–48.63)	12.84 (8.88–19.21)	<0.01
Risk group screening: latent PTB^†^	39.92	15.77	27.15	10.72	
Treatment (per month)					
DS-TB (intensive or continuation phase), 6 months in total	147.70 (84.41–316.61)	58.34 (33.34–125.06)	68.23 (37.46–131.7)	26.95 (14.8–52.02)	<0.01
DR-TB (intensive phase), 7 months max	404.64 (166.95–1058.78)	159.83 (65.95–418.22)	177.53 (97.38–364.65)	70.13 (38.47–144.04)	<0.01
DR-TB (continuation phase), 13 months max	367.34 (145.47–974.24)	145.1 (57.46–384.82)	158.61 (87.69–331.49)	62.65 (34.64–130.94)	<0.01
MDR-TB (first phase), 2 months max	467.33 (276.95–810.36)	184.6 (109.4–320.09)	256.74 (151.82–606.79)	101.41 (59.97–239.68)	<0.01
MDR-TB (second phase), 18 months max	288.62 (162.23–394.96)	114 (64.08–156.01)	141.96 (100.07–204.66)	56.07 (39.53–80.84)	0.25
Inpatient services					
Hospital care (per day)					
DS-TB	62.23 (40.1–102.37)	24.58 (15.84–40.44)	44.85 (19.57–94.18)	17.72 (7.73–37.2)	<0.01
DR-TB	96.56 (79.98–114.2)	38.14 (31.59–45.11)	72.20 (55.15–102.18)	28.52 (21.78–40.36)	0.07

^*^ Excludes TB drugs, Xpert testing, DST, culture, HIV and HCV tests.

† Latent PTB service was only provided in one facility from our sample and statistical test could not be performed.

GEL = Georgian lari; USD = US dollars; PTB = pulmonary TB; DS-TB = drug-susceptible TB; DR-TB = drug-resistant TB; MDR-TB = multidrug-resistant TB; DST = drug susceptibility testing; HCV = hepatitis C virus.

### Adjusted Value TB unit costs and budgeted NTP tariffs for TB interventions

Table 5 presents a comparison of salary-adjusted Value TB TD mean unit costs with the budgeted NTP tariffs for 2020. For outpatient interventions, all TD mean unit costs were higher than the NTP tariffs. For outpatient active PTB screening, the salary-adjusted unit cost was 20% higher than the NTP tariff. For outpatient treatment, the difference between the monthly unit costs and tariffs varied from 50% to 400%, with the largest difference observed for continuation-phase DR-TB treatment. Value TB TD costs were further adjusted for the inflation rates in 2019 and 2020, showing an even higher difference between costs and tariffs.

**Table 5:** Comparison of NTP budgeted tariffs and means of Value TB top-down unit costs for outpatient TB interventions, in 2020 GEL

TB intervention^*^	NTP budgeted tariff (2020 GEL)	Value TB: mean cost adjusted by salary increase (2020 GEL)	Value TB: mean cost adjusted by salary increase and inflated for 2020 (GEL)
Outpatient services			
PTB detection and diagnosis			
Risk group screening: active PTB	52.00	62.14	68.25
Risk group screening: latent PTB†	29.00	43.65	47.94
Treatment (per month)			
DS-TB (intensive and continuation phase), 6 months in total	64.92	166.81	183.20
DR-TB (intensive phase), 7 months max	225.29	469.21	515.34
DR-TB (continuation phase), 13 months max	85.77	429.12	471.31
MDR-TB (first phase), 2 months max	358.00	537.03	589.82
MDR-TB (second phase), 18 months max	140.00	330.70	363.21

^*^ Excludes TB drugs, Xpert testing, DST, culture, HIV and HCV tests.

^†^There was only one observation for latent PTB service.

NTP = national TB control programme; GEL = Georgian lari; PTB = pulmonary TB; DS-TB = drug-susceptible TB; DR-TB = drug-resistant TB; MDR-TB = multidrug-resistant TB; DST = drug susceptibility testing; HCV = hepatitis C virus.

Prior to 2020, the NTP was financing TB inpatient care using differentiated tariffs for Tbilisi and other regions. A comparison of Value TB costs with NTP tariffs (Table 6) shows that there is very little difference in these values for per-day DS-TB treatment in Tbilisi and the other regions. However, the daily NTP tariff for DR-TB exceeds the estimated Value TB unit cost in Tbilisi, but is lower in regional facilities.

**Table 6:** NTP tariffs and Value TB top-down unit costs for inpatient TB interventions, in 2019 GEL

TB intervention^*^		NTP budgeted tariff (GEL)	Value TB unit cost (GEL)	Value TB weighted mean unit cost (GEL)
DS-TB (per day)	Tbilisi	101	103	62.23
	Region	50	52	
DR-TB (per day)	Tbilisi	142	114	96.56^†^
	Region	70	88	

^*^ Excludes TB drugs, Xpert testing, DST, culture, HIV and HCV tests.

^†^Not weighted, all facilities providing inpatient DR-TB services are included in the sample.

NTP = national TB control programme; GEL = Georgian lari; DS-TB = drug-susceptible TB; DR-TB = drug-resistant TB; DST = drug susceptibility testing; HCV = hepatitis C virus.

The comparison of NTP tariffs and Value TB unit costs highlighted the inequity at the sub-national level that arose due to the use of regional differentiation; this prompted a decision to remove differential tariffs and estimate a single common tariff for the central and regional levels. Based on this, weighted mean unit costs were calculated for inpatient care (Table 6).

### TB intervention cost composition

Table 7 presents cost components of the TD salary-adjusted, weighted mean unit costs for outpatient and inpatient services by ownership. Capital costs were higher in public facilities than in private across all interventions. Capital costs for the first phase of MDR-TB treatment was 74% higher in public facilities than private. Staff salary was the unit cost driver for most outpatient services. In general, staff costs in private facilities exceeded those in public facilities with two exceptions, one in active PTB diagnostic service, where staff costs were almost the same, and the second in the intensive phase of MDR-TB treatment, where staff costs in public facilities were higher, possibly due to the high number of MDR-TB patients in the public facility in Tbilisi where higher salaries could be paid. Other recurrent costs as a proportion of total unit cost of interventions ranged from 22% to 46%, with costs in private facilities greater than in public facilities.

**Table 7:** Value TB salary adjusted, weighted mean unit cost components by TB intervention in public and private facilities (top-down), 2020 GEL

TB intervention^*^	Private				Public			
	
	Total unit cost (GEL)	Capital cost^†^ Weighted mean (min-max)	Staff cost^‡^ Weighted mean (min-max)	Other recurrent cost^§^ Weighted mean (min-max)	Total unit cost (GEL)	Capital cost^†^ Weighted mean (min-max)	Staff cost^‡^ Weighted mean (min-max)	Other recurrent cost^§^ Weighted mean (min-max)
Outpatient services								
PTB detection and diagnosis								
Risk group screening: active PTB	62.12	5.73 (2.73–12.31)	26.52 (10.56–41.35)	29.87 (16.13–47.07)	62.24	12.96 (2.83–21.85)	27.8 (21.23–38.53)	21.48 (14.02–38.19)
Risk group screening: latent PTB					43.65	16.66	17.63	9.36
Treatment (per month)								
DS-TB (intensive and continuation phase), 6 months in total	170.82	13.8 (1.98–20.64)	90.39 (30.78–130.68)	66.64 (34.32–126.94)	140.24	24.91 (10.69–44.09)	73.4 (61.99–79.78)	41.94 (21.69–51.79)
DR-TB (intensive phase), 7 months max	484.51	40.12 (6.15–69.29)	256.72 (88.47–370.9)	187.67 (104.22–359.75)	368.01	75.52 (36.47–115.04)	180.2 (133.85–221.25)	112.28 (55.97–146.95)
DR-TB (continuation phase), 13 months max	450.09	37.42 (5.9–68.5)	230.97 (80.75–332.98)	181.71 (104.02–336.4)	290.43	54.61 (29.36–89.6)	147.76 (120.76–166.65)	88.07 (41.82–110.07)
MDR-TB (first phase), 2 months max	487.16	52.46 (19.15–69.66)	261.42 (168.46–325.91)	173.29 (118.53–249.09)	845.29	224.85	307.01	313.52
MDR-TB (second phase), 18 months max	342.37	37.18 (13.46–52.11)	178.32 (116.99–212.44)	126.86 (99.18–173.27)	258.60	54.46	106.67	97.44
Inpatient services								
Hospital care (per day)								
DS-TB	64.30	9.57	16.04	38.68	60.86	11.87 (8.29–19.02)	21.17 (21.09–21.21)	27.82 (20.74–31.35)
DR-TB	79.98	9.65	27.09	43.25	104.85	22.23 (10.81–33.66)	47.05 (38.33–55.77)	35.57 (32.86–38.27)

^*^ Excludes TB drugs, Xpert testing, DST, culture, HIV and HCV tests.

^†^ Capital cost: buildings; laboratory and medical equipment; other equipment; furniture; vehicles; training.

^‡^ Staff cost: clinical and support staff.

^§^ Other recurrent cost: medical supplies; other (non-medical) supplies; capital maintenance; utilities; fuel and other transport recurrent (including maintenance and courier services); food, supplements, including food service; and other recurrent.

GEL = Georgian lari; PTB = pulmonary TB; DS-TB = drug-susceptible TB; DR-TB = drug-resistant TB; MDR-TB = multidrug-resistant TB; DST = drug susceptibility testing; HCV = hepatitis C virus.

For per-day inpatient DS-TB treatment costs, there was a small difference between public and private settings, with slightly (5%) higher costs in private facilities. Other recurrent cost was a cost driver in both settings. Due to the differentiated staff salaries between Tbilisi and at the sub-national level, salaries were lower for both DS-TB and DR-TB treatment per day in the private facility, which in our sample comprised only one facility outside Tbilisi. The per-day cost of DR-TB treatment in public facilities is about 30% higher than private facilities. Capital costs were approximately twice as high in public facilities, as buildings and equipment were more expensive in the capital city.

## DISCUSSION

The Value TB study assessed BU and TD unit costs of TB interventions. Measuring costs using both methods is recommended, as these provide valuable information for policy and planning by informing managers about current levels of efficiency.^9^,^11^ BU costs are based on detailed measurement of all resources used for specific health interventions, and although the approach captures some inefficiencies in processes, a TD cost analysis uses a more holistic approach and is therefore able to highlight capacity inefficiency.^9^ Our study showed that TD costs were greater than BU estimates, with varying differences (between 34% and 132%), depending on the TB intervention (the largest difference was noted for DR-TB interventions), suggesting some excess capacity in TB clinics in Georgia.

Previous NTP budgeting in Georgia was based on a BU approach for tariff setting, but included only a selected number of ingredients. Specifically, the outpatient voucher was constructed based on service unit costs collected from service providers, protocol-recommended service quantity and fixed minimum salaries for TB specialists defined by the NTP. Prior to 2020, the inpatient daily tariff was also based on estimates from inpatient providers. The use of Value TB TD estimates to inform policy discussions around TB financing models was based on a desire to incorporate more realistic costs, particularly for services with low patient volumes due to low demand in those catchment areas. As expected, estimated costs for outpatient interventions exceeded the NTP tariff, particularly for outpatient treatment. This difference is partly explained by inefficiency such as staff downtime in facilities with small numbers of patients (mainly private) or higher capital costs in public facilities with more expensive infrastructure (equipment and buildings); this is demonstrated by the comparison of cost categories between public and private facilities. Capital costs for first-phase MDR-TB treatment was, however, found to be higher for public providers. This could be explained by the switch to a new drug regimen for MDR-TB patients receiving care in the capital city in 2018. Although drug costs were excluded from service costs, this regimen requires more intensive clinical monitoring, including tests and instrumental investigations.

By and large, we found that the unit costs of services in private facilities were higher than public facilities. In Georgia, approximately 80% of all facilities providing outpatient services are privately owned and located at the sub-national level. Public providers are mostly concentrated in the capital, Tbilisi, and other major cities. These public sites serve relatively large number of patients, partially explaining their lower costs. There is little interest among private providers to participate in TB service provision, as they see no commercial benefit at a low price, but there are few alternative service providers in many geographic areas with lower patient volumes.

Following privatisation of health care provision in 2012, all service providers were obliged to retain TB programme services until 2018.^5^ Post 2018, the state negotiated the terms for TB service provision with the private sector by conditional participation in the Universal Health Care (UHC) programme. In addition, to boost their participation in TB service provision, GeneXpert machines and cartridges were provided free of charge to some private providers for use in TB diagnostics and infection control activities. The 30–35% salary increase defined by the NTP was also implemented as a means of motivating TB doctors and nurses. These and other regulatory measures ensured uninterrupted delivery of quality TB services countrywide; however, in the context of fragmented service delivery between the UHC programme and the NTP, and much lower payment for outpatient services may push private providers to retreat from TB service delivery if they are not able to cover their costs with the latest tariffs.

Circumstances relating to inpatient services are different. The per-day NTP financing of DR-TB treatment exceeded actual costs in Tbilisi, but is lower in the regions. It is worth mentioning that the cost components of NTP inpatient tariffs and Value TB unit costs are not identical. This is partly because the NTP tariff includes surgical interventions, while Value TB does not. Surgical interventions occur mostly in Tbilisi and are largely for DR-TB patients, comprising approximately 17% and 4% of the Tbilisi DR and DS-TB inpatient treatment tariffs, respectively. Even after removing the cost of surgical interventions from the NTP tariff, the latter still exceeded the Value TB unit cost for DR-TB treatment in the capital.

The Value TB cost study was an important input into the policy discussions on provider payments. Policy discussions focused mainly around inpatient costs, specifically how to increase efficiency of inpatient services using new payment models without compromising service quality. Different options were discussed, among which diagnosis-related groups (DRGs) were prioritised as one of the effective cost-containing mechanisms in hospital financing. DRG payments focus on technical efficiency to make better use of available resources and reduce average length of hospital stay, but they also encourage hospitals to increase the number of patients served.^12^

As part of these discussions, a decision was made to move from payment per day to DRG payment for inpatient services, which was informed by the availability of having a reliable estimate of Value TB unit costs for inpatient DS- and DR-TB services. The change has been in place since January 2020. While DRG brings a risk of increased hospitalisation, the country is moving towards patient-centred care, which is expected to facilitate treatment initiation at the outpatient level for more TB patients. Without further motivating outpatient care providers and reducing the gap between financing and costs, it will be challenging to succeed in this directive; an understanding of provider costs can inform these future policy decisions.

Finally, our estimates for the full first-line treatment is closer to the respective cost for lower-middle-income rather than for upper-middle-income economies to which Georgia belongs.^13^ Georgia has historically been classified as lower-middle-income country and upgraded in 2018, with its gross national income per capita only slightly exceeding the threshold.^14^ Siapka et al. also found a positive association between unit cost and country income;^13^ therefore, with economic growth, TB costs are likely to increase in Georgia.

The study had several limitations. To overcome data availability issues, such as managerial staff salaries in private facilities, assumptions were made based on expert consultation. As TB monitoring visits were not registered in the facilities, calculations were based on the treatment protocol, rather than on observation or record. Observations of certain practices, including diagnostic, monitoring and DOT visits, were not possible for ethical reasons; interviews were therefore conducted, which may be subject to reporting bias. Finally, Value TB intervention unit costs did not include surgery and invasive intervention-related costs occurring at the central level, which occurs in approximately 30% of all TB hospital admissions.
